# An Olive Oil Mill Wastewater Extract Improves Chemotherapeutic Activity Against Breast Cancer Cells While Protecting From Cardiotoxicity

**DOI:** 10.3389/fcvm.2022.867867

**Published:** 2022-04-14

**Authors:** Nadia Benedetto, Luana Calabrone, Karolina Gutmańska, Nicoletta Macrì, Maria Grazia Cerrito, Riccardo Ricotta, Giuseppe Pelosi, Antonino Bruno, Douglas M. Noonan, Adriana Albini

**Affiliations:** ^1^IRCCS MultiMedica, Milan, Italy; ^2^School of Medicine and Surgery, University of Milano-Bicocca, Monza, Italy; ^3^IRCCS MultiMedica, Sesto San Giovanni, Italy; ^4^Department of Oncology and Hemato-Oncology, University of Milan, Milan, Italy; ^5^Immunology and General Pathology Laboratory, Department of Biotechnology and Life Sciences, University of Insubria, Varese, Italy; ^6^European Institute of Oncology (IEO) IRCCS, Milan, Italy

**Keywords:** chemotherapy, breast cancer, polyphenol, olive, cardiomyocyte

## Abstract

Cardiovascular toxicity in cancer patients receiving chemotherapy remains one of the most undesirable side effects, limiting the choice of the most efficient therapeutic regimen, including combinations of different anticancer agents. Anthracyclines (doxorubicin) and antimetabolites (5-fluorouracil (5-FU), capecitabine) are among the most known agents used in breast cancer and other neoplasms and are associated with cardiotoxic effects. Extra-virgin olive oil (EVOO) is rich in polyphenols endowed with antioxidant cardioprotective activities. Olive mill wastewater (OMWW), a waste product generated by EVOO processing, has been reported to be enriched in polyphenols. In this study, we investigated the activities of polyphenol-rich extract from OMWW, A009, in cooperation with chemotherapy on two breast cancer cell lines, namely, BT459 and MDA-MB-231, in a cardio-oncology perspective. The effects of A009 on cardiac cells were also investigated with and without chemotherapeutic agents. Cell viability was determined on BT459 and MDA-MB-231 (i.e., breast cancer cells) and H9C2 (i.e., rat cardiomyocytes) cells, using 3-(4,5-dimethylthiazol-2-yl)-2,5-diphenyltetrazolium bromide (MTT) assay. A spheroids assay was used as a 3D *in vitro* model on BT459 and MDA-MB-231 cells. For *in vivo* studies, the murine sponge assay of angiogenesis was used as a model of breast cancer-associated vascularization. The embryo of *Danio rerio* (zebrafish) was used to detect the cardioprotective activities of the OMWW. We found that the A009 extract exhibited antiangiogenic activities induced by breast cancer cell supernatants and increased T-cell recruitment *in vivo*. The combination of the OMWW extracts with doxorubicin or 5-FU limited BT459 and MDA-MB-231 cell viability and the diameter of 3D spheroids, while mitigating their toxic effects on the rat H9C2 cardiomyocytes. Cardioprotective effects were observed by the combination of OMWW extracts with doxorubicin in zebrafish embryos. Finally, in human cardio myocytes, we observed 5-FU-induced upregulation of the inflammatory, senescence-associated cytokine IL6 and p16 genes, which expression was reduced by OMWW treatment. Our study demonstrates that the polyphenol-rich purified OMWW extract A009 combined with cancer chemotherapy could represent a potential candidate for cardiovascular protection in breast cancer patients, while increasing the effects of breast cancer chemotherapy.

## Graphical Abstract

The cartoon summarized the major approaches and insights of the manuscript: **(A)** the possibility to recover waste material from extra-virgin olive oil (EVOO) processing that allows the repurposing of polyphenol-rich extracts characterized by **(B)** antiangiogenic activities *in vivo*, antiproliferative activities *in vitro* on BC cell lines **(C)**, cardioprotective activities on rat and human cardiomyocytes (HCMs) **(D)**, and *in vivo* on the zebrafish embryo heart **(E)**. 
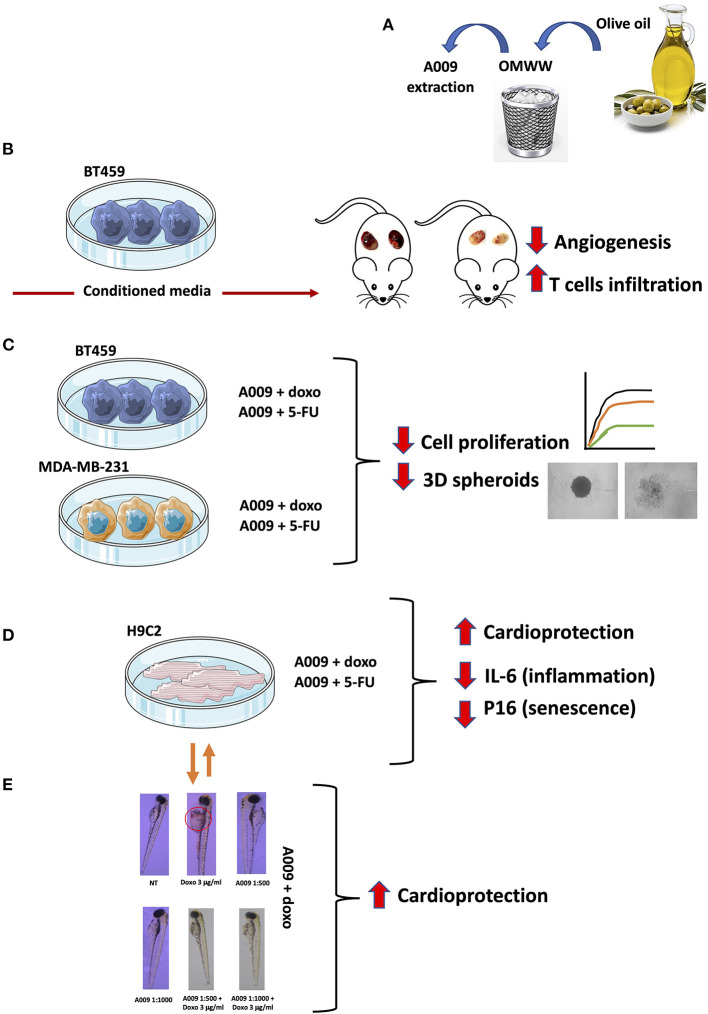

## Introduction

Together with cardiovascular diseases, cancer still accounts as a major cause of death in the world (1, 2). Breast cancer (BC) is the fifth most prevalent cause of cancer death worldwide and is the most common malignancy among women (3). BC is characterized by the presence of several different subtypes, which are generally classified as hormone receptor (HR)-positive and human epidermal growth factor receptor 2 (HER2)-overexpressing BCs or triple negative BC. The presence of the markers has allowed the development of targeted therapies. Tumors without the expression of HR or HER2 are classified as triple-negative BC (TNBC). TNBC has a higher mortality rate than HR-positive or HER-2-overexpressing BC because of its high recurrence rate and metastatic potential (4).

Among the current strategies for the treatment of TNBC, chemotherapy represents the major option (5, 6). Taxanes (e.g., paclitaxel and docetaxel) and anthracyclines (e.g., doxorubicin and epirubicin) are usually the main choice (7), in combination with platinum (e.g., carboplatin), antimetabolites [e.g., capecitabine, similar to 5-fluorouracil (5-FU)] and/or alkylating agent (e.g., cyclophosphamide); however, their possible toxicity, low aqueous solubility, and rapid *in vivo* clearance can represent a limit (7, 8). All these agents are often used in combination with targeted therapy.

Despite the great medical and pharmaceutical advances, these limitations remain a big challenge. According to the Food and Drug Administration (FDA), about 1 million of severe adverse drug events (ADEs) were reported in only 1 year in the United States, including death (9). Chemotherapy-induced cardiotoxicity is one of the most common ADEs and can cause more or less serious manifestations, such as changes in ECG, arrhythmia, bradycardia, tachycardia, and heart failure, which lead to an increase in morbidity and mortality (7, 9). In this complex contest, prevention still remains the most promising approach for all types of cancer. Natural products or agents derived from foods and beverages (or their synthetic analogs) have been found to exert antitumor and tumor-preventive activities in diverse preclinical settings, and some are currently employed in the clinic. Among them, phenolic compounds have gained the attention of the scientific community, thanks to their plethora of beneficial effects on health, that include antibacterial, anti-inflammatory, and anticancer activities. Also, several preclinical studies, including those published by our research group, showed that diverse phytocompounds, while synergizing with chemotherapeutic drugs, have cardioprotective activity (10, 11).

Southern European countries have lower incidence of cancer and cardiovascular disease than northern European countries or the United States. The Mediterranean diet has been proposed as the main protective factor for this benefit (12). In this context, angioprevention is an important concept to consider, since angiogenesis prevention through bioactive compounds present in the Mediterranean diet components could explain in part the chemopreventive effect of this diet model in cancer (13–17). Extra-virgin olive oil (EVOO) is a major component of the Mediterranean diet, with numerous beneficial effects, which concern the ability to prevent diseases that can be linked to oxidative damage, such as neurodegenerative diseases, cancer, and cardiovascular diseases (18). EVOO protective role is due to its enriched content in phytochemicals: the main fraction (95–97%) is the lipophilic one, which is represented by both monounsaturated and polyunsaturated fatty acids (i.e., omega-3 and omega-6) (19). The polar fraction is mainly represented by polyphenolic compounds like oleuropein, tyrosol, and hydroxytyrosol, which possess strong anti-inflammatory and antioxidant properties (20). A major issue within the industrial processing of EVOO is the generation of large amount of liquid waste product, including, olive mill wastewater (OMWW) (21, 22). The high content of pollutants within the waste requires special disposal and cost-effective procedures that significantly impact both the health environment and the industrial management. In contrast, it has been found that OMWW is rich in polyphenolic compounds, endowed with antibacterial and antioxidant activities, thus representing a valid product to be considered in scientific research (23).

We previously reported that the A009 polyphenol-rich extracts, purified form OMWW, exhibit chemopreventive and angiopreventive properties, *in vitro* and *in vivo*, in different cancer types (e.g., lung, colon, and prostate cancer cells) and endothelial cells (11, 24–27).

In this study, we investigated the A009 effect on tumor growth of BC cells, alone or in combination with a chemotherapeutic agent. In addition, we examined the potential A009 cardioprotective activity, against chemotherapy-induced cardiovascular damages, using both *in vitro* and *in vivo* models (i.e., *Danio rerio* and *Mus musculus*). In this study, we focused on doxorubicin and 5-FU, which are very prominent, highly active anticancer drugs, however, endowed with cardiotoxic effects. Work flow is described in the Graphical Abstract.

## Materials and Methods

### Chemicals

5-Fluorouracil was purchased from Sigma-Aldrich and was dissolved in dimethyl sulfoxide (DMSO) and used for *in vitro* experiments as detailed below. Doxorubicin hydrochloride (Doxo) was purchased from Abcam and was dissolved in Milli-Q water. 3-(4,5-dimethylthiazol-2-yl)-2,5-diphenyltetrazolium bromide (MTT) was purchased from Sigma-Aldrich. The synthetic hydroxytyrosol (Hyt), ≥98% in purity, was purchased from Cayman Chemicals (Ann Arbor, MI, USA). Hyt was dissolved in ethanol (EtOH). DMSO and EtOH vehicles were used as controls.

### Preparation of A009 Extracts

Olive oil mill wastewaters were kindly provided by Agriturismo Fattoria La Vialla (Castiglion Fibocchi, Arezzo, Italy) and used to obtain the phenol-rich purified extract A009 (Patent No. 1420804; No. 1420805). The experiments were performed using the extract A009. The extraction procedures of A009 obtained from OMWW and its polyphenol content have been previously described (24). The polyphenol content of the A009 extract is shown in Supplementary Table 1.

### Cell Line Culture and Maintenances

The human metastatic BC cells, e.g., MDA-MB-231 (purchased from ATCC), were maintained in the Dulbecco's Modified Eagle's Medium (Gibco-BRL) supplemented with 10% fetal bovine serum (FBS) (Euroclone), 2 mM L-glutamine (Euroclone), 100 U/ml penicillin, and 100 μg/ml streptomycin (Euroclone), at 37°C, 5% CO_2_. The human BC cell line BT549 (purchased from ATCC) was grown in Roswell Park Memorial Institute (RPMI) 1640 supplemented with 10% FBS, 2 mM L-glutamine, 100 U/ml penicillin and 100 μg/ml streptomycin, and 0.023 U/ml insulin at 37°C, 5% CO_2_. The human cardiomyocytes (HCMs, purchased by PromoCell) cells were maintained in the Myocyte Growth Medium (PromoCell) plus Myocyte supplements mix (PromoCell), in addition to 10% FBS, 2 mM L-glutamine, 100 U/ml penicillin, and 100 μg/ml streptomycin, at 37°C, 5% CO_2_. The rat cardiomyocytes cell line H9C2 (purchased by PromoCell) was maintained in DMEM-F12, supplemented with 10% FBS, 2 mM L-glutamine, 100 U/ml penicillin, and 100 μg/ml streptomycin. All the cell lines used in the study were routinely checked for eventual mycoplasma contamination, before being used.

### Generation of Conditioned Media

Conditioned media (CM), for subsequent *in vivo* studies, was obtained from the BT459 BC cell line. Briefly, 3 × 10^6^ BT459 were seeded into t100 Petri dishes (Corning) in RPMI 1640 supplemented with 10% FBS, 2 mM L-glutamine, 100 U/ml penicillin and 100 μg/ml streptomycin, and 0.023 U/ml insulin at 37°C, 5% CO_2._ When cells reached 80% of confluency, cells were starved for 48 h in 10 ml serum-free RMPI medium. Finally, CM were collected, residual cells and debris were discarded, by centrifugation, and concentrated with Concentricon devices (Millipore, Temecula, CA) with a 5 kDa membrane pore cutoff.

### *In vivo* Angiogenesis Sponge Assay as a Model of Breast Cancer-Associated Angiogenesis

To use a rapid model of breast cancer-associated angiogenesis *in vivo* (28–30), the ability of A009 (1:250) to inhibit vascularization, induced by CM of BC cell lines, was investigated using the UltiMatrix (Biotechne) Matrigel sponge assay in mice. Briefly, liquid UltiMatrix (10 mg/ml) was mixed with 50 μg of total protein BT459-concentrated CM, alone or in combination with the A009 extract (1:250 dilution) and inoculated in cold liquid form, which polymerizes *in vivo*. UltiMatrix alone or UltiMatrix supplemented with a cocktail of proangiogenic factors VEGF, TNFα and Heparin (VTH) (100 ng/ml vascular endothelial growth factor (VEGF)-A, 2 ng/ml tumor necrosis factor (TNF)-α, and 25 U/ml heparin) were used as negative and positive controls, respectively. Each mixture was brought to a final volume of 0.6 ml and injected subcutaneously into the right and left flanks of 6–8-week-old C57/BL6 female mice (Charles River Laboratories, Calco (Lecco), Italy) with a cold syringe. All animals were housed in a conventional animal facility with 12 h light/dark cycles and fed *ad libitum*. Manipulation of animals was performed in accordance with the Italian and European Community guidelines (D.L. 2711/92 No.116; 86/ 609/EEC Directive), the 3 R's declaration, and approved by the institutional ethics committee. All the procedures applied were approved by the local animal experimentation ethics committee (ID# #06_16 Noonan) of the University of Insubria and by the Health Ministry (ID#225/2017-PR).

Groups of 3–7 mice were used for each treatment. At body temperature, the UltiMatrix polymerizes to a solid gel and becomes vascularized in response to angiogenic substances. Four days following injection, the gel plugs were recovered and divided into two parts. One half was formalin-fixed, paraffin-embedded to generate paraffin blocks processed for histological analysis; the other half from gel plugs was minced and diluted in water to measure the hemoglobin content with a Drabkin's Reagent Kit (Sigma-Aldrich), and part was mechanically processed for the subsequent flow cytometry analysis.

### Immunohistochemistry Analysis of Utimatrix Sponges

All the processing for the immunohistochemistry analysis on the Utimatrix sponges were performed by the Unit of Pathological Anatomy, IRCCS MultiMedica, Milan, Italy, by a routine system on an automated immunostainer (BenchMark ULTRA IHC/*in situ* hybridization System, Ventana-Roche Group, Basel, Switzerland). Hematoxylin and Eosin-stained sections were used to acquire micrographs, at 40× magnification.

### Flow Cytometry Analysis for Cell Infiltrate in the UltiMatrix Plugs

Part of the recovered UltiMatrix plugs were mechanically processed by scissors, then placed into 70 mm cell strainers (BD Biosciences), and pressured with a syringe swab. The cell suspension obtained was used for the flow cytometry analysis to detect the immune cell infiltrate. Cells were stained for 30 min at 4°C, at dark, with the following fluorophore-conjugated antimouse antibodies, all purchased from BD Biosciences: CD45-BUV395, F4/80-PECF594, CD3e-BB700, and NK1.1-BV650. For fluorescence-activated cell sorting (FACS) analysis, viable cells were gated according to physical parameters (FSC/SSC). Following the gating of CD45^+^ cells, immune cells were identified as follows: CD45^+^:F4/80^+^ cells (macrophages), CD3^+^ cells (total T cells), and CD3^−^NK1.1^+^ cells (total NK cells).

### Assessment of Combination Effect of Chemotherapy and A009 on Breast Cancer Cell Lines and Rat Cardiomyocytes by MTT Assay

To investigate whether the A009 extract could synergize with chemotherapy, cell viability of the BC cell lines MDA-MB-231 and BT549 was evaluated by the MTT assay (Supplementary Table 2). The 2 × 10^3^ cells of BT549 and MDA-MB-231 BC cell lines were seeded in 96-well plates and, after adhesion, treated with A009 extract (dilution 1:800) and Hyt (dilution 1:800) for 24 h. The medium was then substituted with the chemotherapy drug 5-FU 100 μM or Doxorubicin 1 μM, alone or in combination with A009 or Hyt for 48 and 72 h. At each time point, media were replaced with fresh complete medium, supplemented with 0.5 mg/ml MTT reagent, and then incubated for 3 h at 37°C with 5% CO_2_. This colorimetric assay is based on the reduction of a yellow tetrazolium salt (3-(4,5-dimethylthiazol2-yl)-2,5-diphenyltetrazolium bromide or MTT) to purple formazan crystals by metabolically active cells. The viable cells contain NAD(P)H-dependent oxidoreductase enzymes that reduce the MTT to formazan. MTT was removed, and formazan crystals were dissolved using 100% DMSO. The darker the solution, the greater the number of viable, metabolically active cells. The absorbance was recorded at 570 nm wavelength with the microplate spectrophotometer SpectraMax M2 (Molecular Devices, Sunnyvale, CA). To evaluate the effects of the A009 extracts on chemotherapy-induced cardiotoxicity, the same experiment was repeated on rat cardiomyocytes H9C2 cells (Supplementary Table 2).

### Generation of Tumor Spheroids

We investigated the capability of the A009 extract to synergize with chemotherapy, in reducing the generation of BC tumor spheroids. BT459 or MDA-MB-231 cells were cultured in 20 μl hanging drops at a density of 4 × 10^3^ cells/drop, in complete RPMI medium, with or without treatment. After spheroids formation, which typically occurs within 24 h, the spheroids were transferred to 96-well plates, containing 100 μl/well of fresh culture medium with or without treatment (one spheroid/well), previously coated with 2% agar and growth on the bottom of 60 mm tissue culture dish, at 37 °C and 5% CO_2_. BT459 and MDA-MB-231 spheroids were treated with the A009 extract + 5-FU 100 μM combination, or the A009 extract + Doxo 1 μM combination, or A009 extract or 5-FU alone. Growth of the spheroidal colonies was monitored for the following days, replacing culture medium and treatments, with fresh ones, each 48 h. Images were acquired at 3, 6, and 12 days, following spheroid generation and treatments. Untreated and treated spheroids' area was measured, for each time point, using the ImageJ software and normalized for the respective area at day 3. The diameter was calculated based on the spheroid area, using the d = √(4A/π) formula. Finally, the average area, between treatment groups, was compared.

### *In vivo* Studies on Zebrafish Embryos

To evaluate the cardioprotective effect of the A009 extract, we used the embryos of *Danio rerio* (zebrafish), a robust animal model for cardiovascular diseases (31–33). Zebrafish eggs were incubated at a temperature of 26 ± 1°C, in a 12:12 h light:dark regime. Developmental stages were identified according to Kimmel et al. (34). Eggs were collected and washed two times with ISO-water (80 mM CaCl_2_, 20 mM MgSO_4_, 30.8 mM NaHCO_3_, and 3.09 mM KCl) according to DIN ISO 150888 (International Organization for Standardization (ISO), 2009). All experiments were performed on zebrafish embryos exposed to the indicated agents for 24, 48, and 72 h post fertilization (hpf). Ten fertilized eggs were maintained in 24-well plates, with a proportion of 1 embryo/2 ml of solution. Embryos received A009 (dilutions 1:1,000 or 1:500), alone or in combination with the cardiotoxic agent doxorubicin (3 μg/ml) or left untreated. Embryo development was monitored at 48 and 72 hpf using an inverted stereomicroscope (Leica), by tracing the development of eyes, heartbeat, blood circulation, pigmentation, body shape malformations, edemas, detachment of the tail, and delay in development. The effect on embryo viability was determined by counting the number of dead embryos per experimental condition. Several parameters to trace the treatment induced congenital embryo abnormalities were monitored as listed in Supplementary Tables 3, 4. Congenital embryo abnormalities monitored included ischemia in the yolk sack (IS-YS), malformation of the heart (M.HT), ischemia in the tail (IS-TA), malformation of the tail (MT), yolk sack malformation (YS-DE), swim bladder malformation (SWB-DE), pericardial edema (PE), and ischemia in the brain (IS-BR).

### Quantitative Real-Time PCR

Total RNA was extracted from HCM exposed to A009 (dilution 1:800) alone or in combination with 5-FU 100 μM for 24 h. The TRIzol method was used, following separation with chloroform precipitation of RNA with isopropanol (Sigma-Aldrich). The RNA pellet was washed twice with 75% ethanol (Sigma-Aldrich) and resuspended in nuclease-free water. RNA concentration was determined using the Nanodrop Spectrophotometer ND-1000 (Thermo Fisher Scientific). Reverse transcription was performed using the SuperScript VILO cDNA synthesis kit (Thermo Fisher Scientific), starting from 1,000 ng of total RNA. Quantitative real-time PCR was performed using SYBR GreenMasterMix (Applied Biosystems) on the QuantStudio 6 Flex RealTime PCR System Software (Applied Biosystems). All reactions were performed in duplicate. The relative gene expression was indicated as relative to nontreated cells, normalized to the housekeeping gene 18S. IL-6 (Fw-*AGACAGCCACTCACCTCTTCAG*, Rv-*TTCTGCCAGTGCCTCTTTGCTG*), p16 (Fw-C*TCGTGCTGATGCTACTGAGGA*, Rv-*GGTCGGCGCAGTTGGGCTCC*), and the housekeeping 18S (Fw-*CGCAGCTAGGAATAATGGAATAGG*, Rv *CATGGCCTCAGTTCCGAAA*) primers, for qPCR, were designed using the NCBI Primer BLAST tool and purchased from Integrated DNA Technologies (IDT, Coralville, IA, USA).

### Statistical Analysis

The statistical significance between multiple datasets was determined using the GraphPad Prism software v9. Flow cytometry data were analyzed using the FlowJo software, v10. Data are expressed as means ± SEM, one-way ANOVA, followed by the Tukey's *post-hoc* test. The *p* ≤ 0.05 were considered statistically significant.

## Results

### A009 Inhibits Angiogenesis *in vivo*

**Figure 1:**
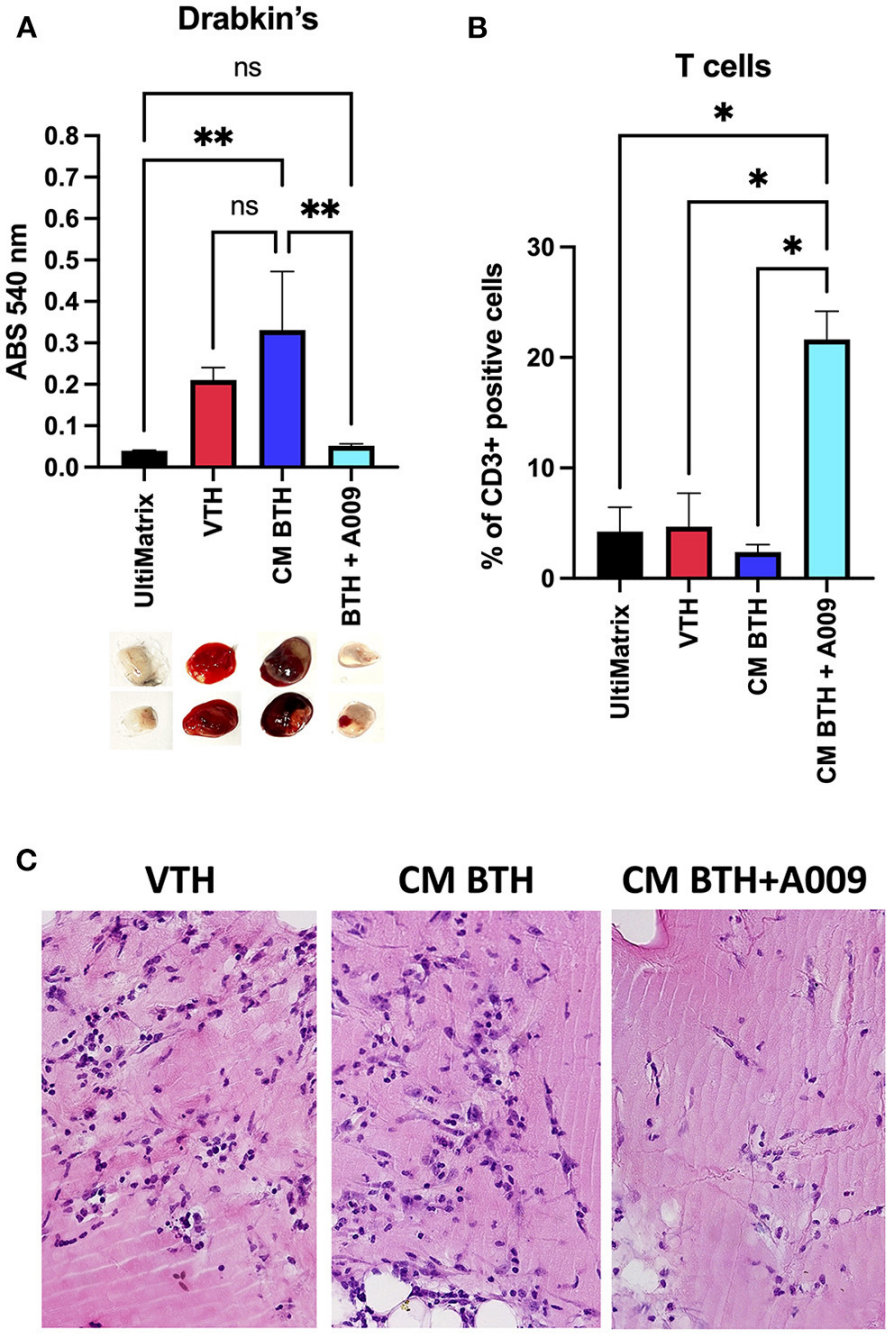
Effects of the A009 extract on angiogenesis and immune cell infiltration *in vivo*. The effects of the A009 on angiogenesis, induced by CM of BC cell lines, and immune cell infiltration *in vivo*, was evaluated by the UltiMatrix Matrigel sponge assay. **(A)** Determination of the hemoglobin content in the excised pugs, using the Drabkin's assay and visual inspection of excised pellets; **(B)** determination of T-cell infiltration, detected as CD3^+^ cells, in the plugs by flow cytometry. Data are shown as mean ± SEM, one-way ANOVA, *p < 0.05 and **p < 0.01. VTH (vascular endothelial growth factor (VEGF), tumor necrosis factor (TNF)-α, heparin), CM BTH (CM BT549 + heparin), A009 extract. Hemoglobin is decreased by A009 and T-cell infiltrates are increased. **(C)** Histological examination of pellets. Sections of paraffin embedded plugs were stained with hematoxylin eosin. VTH: VEGF-TNF-α-heparin containing UltiMatrix show angiogenesis. Pellets with BTH conditioned media show angiogenesis with many vessels. Pellets with A009 in addition to BTH CM show reduced angiogenesis with few vessels.

To evaluate the effect of the extract A009 (dilution 1:250) on angiogenesis, a hallmark of cancer, induced by CM of BC cells *in vivo*, a Matrigel sponge assay was performed in C57/BL6 female mice. We found that the A009 extract (1:250) was able to reduce the angiogenic activities exerted by the BT549 BC CM, as revealed by the colorimetric analysis of the excised UltiMatrix plugs (Figure 1A). The A009 extract (1:250) was able to reduce the total hemoglobin content in the treated plugs, as compared with those containing the BT549 BC CM alone, in a statistically significant dependent manner (Figure 1A). Proangiogenic recruitment of endothelial cells was also decreased as demonstrated by histological staining (Figure 1C). Given the immune-modulatory properties of the A009 extract, we tested its ability to enhance immune cell number in the exposed sponge, following excision. We found that the A009 extract (1:250) was able to increase the infiltration of T cells in treated plugs, as compared with those containing the BT549 BC CM alone; in a statistically significant manner (Figure 1B), macrophages and NK cells are non-significantly modified (Supplementary Figure 1).

### Effect of A009 on Cell Viability of BC Cell Lines in 2D and 3D Models *in vitro*

**Figure 2:**
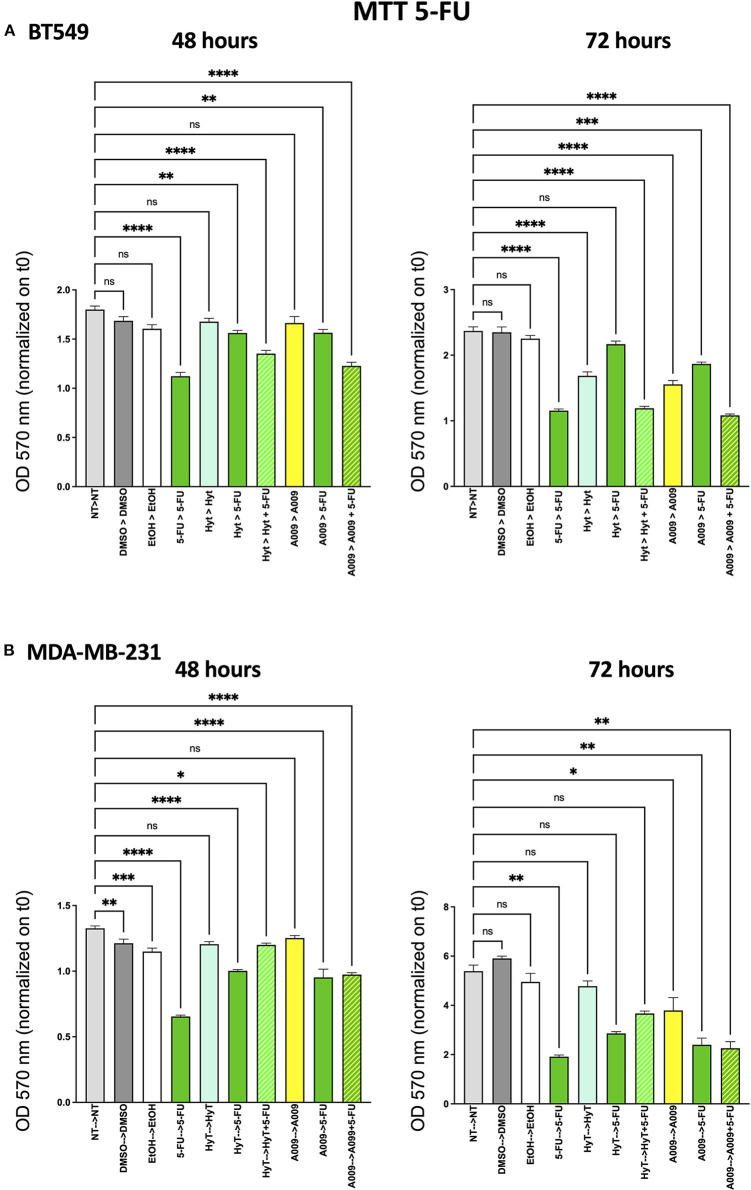
Effects of the A009 extract in combination with 5-fluorouracil (5-FU) on breast cancer cell lines. BT549 **(A)** and MDA-MB-231 **(B)** cells were pretreated with extract A009 (dilution 1:800) or hydroxytyrosol (Hyt) (same concentration as that present in the 1:800 diluted A009 extract), for 24 h. Later, the medium was replaced with 5-FU 100 μM alone or in combination with A009 or Hyt for 48 and 72 h. Proliferation was detected by the MMT assay at the indicated time points. The experiments were performed in quadruplicate and repeated two times. Results are expressed as the mean of the absorbance normalized on the T0 ± SEM, one-way ANOVA, *p < 0.05, **p < 0.01, ***p < 0.001, and ****p < 0.0001. A009 activity was enhanced by addition of chemotherapy towards breast cancer cells.

**Figure 3:**
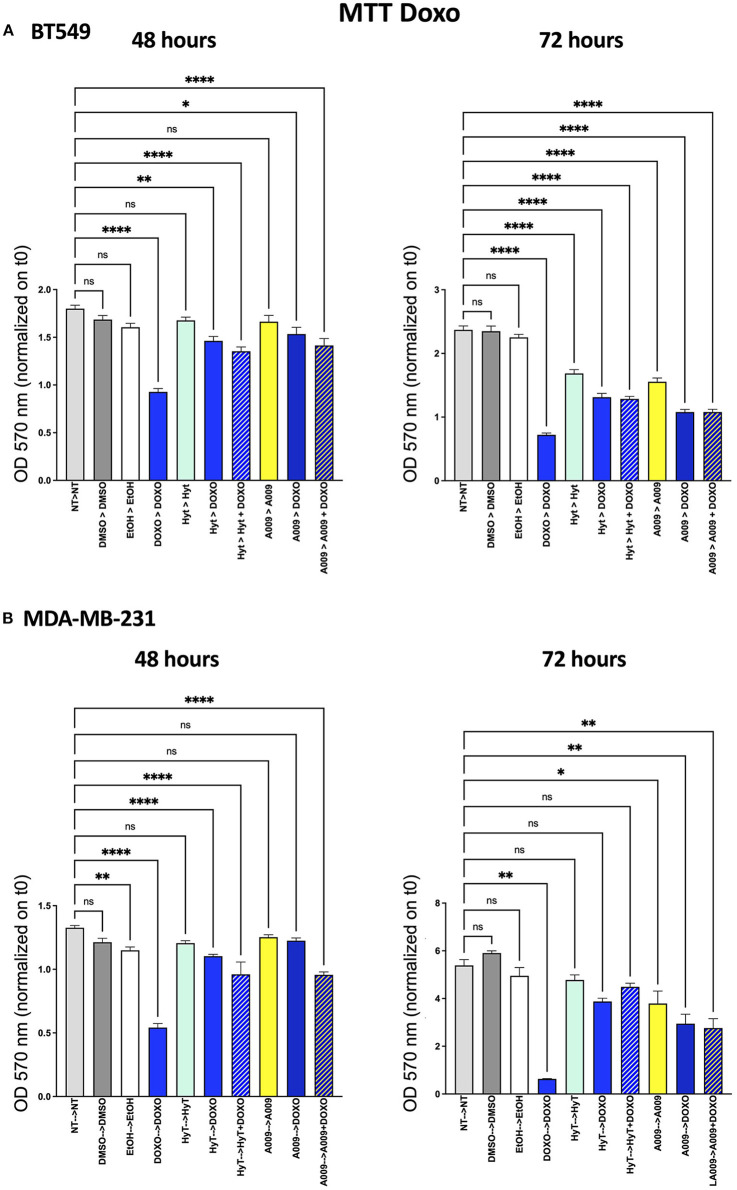
Effects of the A009 extract in combination with doxorubicin on breast cancer cell lines. BT549 **(A)** and MDA-MB-231 **(B)** cells were pretreated with extract A009 (dilution 1:800) or Hyt (same concentration as that present in the 1:800 diluted A009 extract), for 24 h. Later, the medium was replaced with doxorubicin 1 μM alone or in combination with A009 or Hyt for 48 and 72 h. Proliferation was detected by the MMT assay at the indicated time points. The experiments were performed in quadruplicate and repeated two times. Results are expressed as the mean of the absorbance normalized on the T0 ± SEM, one-way ANOVA, *p < 0.05, **p < 0.01, and ****p < 0.0001. A009 activity was enhanced by addition of chemotherapy toward breast cancer cells.

**Figure 4:**
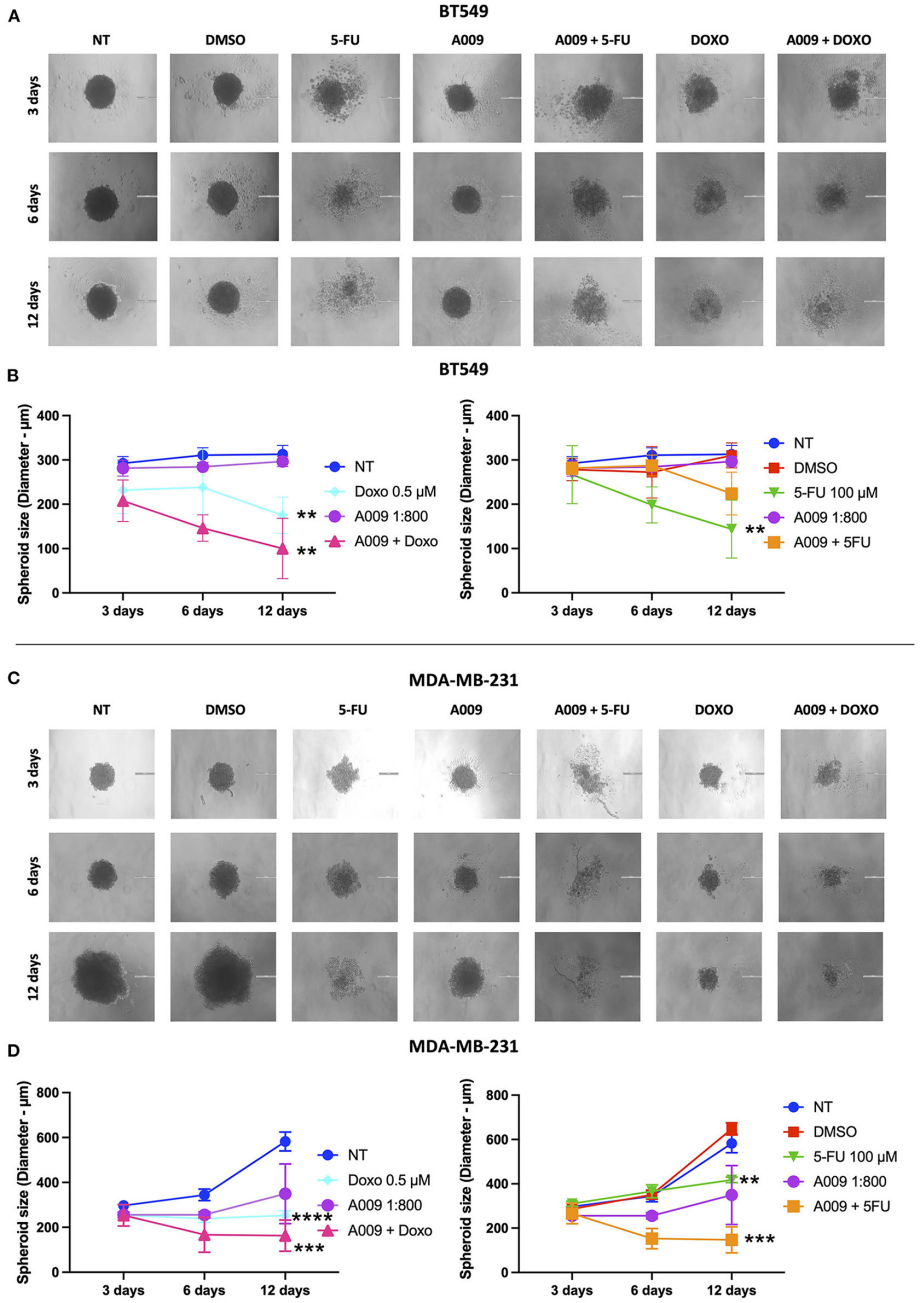
Effects of the combination A009+chemotherapy on breast cancer spheroids. Single spheroids were generated by culturing 4 x 10^3^ BT549 or MDDA-MB-231 cells in nonadherent conditions. BT459 **(A,B)** and MDA-MB-231 **(C,D)** spheroids were treated with the combination of A009 extract + 5-FU or Doxo, A009 or drugs alone, for 3, 6, and 12 days. During the treatment kinetic, spheroid diameters were detected, and spheroid macrophotographs were captured. The experiments were performed in quadruplicate and repeated two times. Scale bar = 200 μm. Data are shown as mean ± SEM, two-way ANOVA, **p < 0.01, ***p < 0.001, and ****p < 0.0001. A009 combinations with chemotherapy reduced size of breast cancer cell spheroids.

The effect of the A009 extract on tumor cell viability was investigated using the MTT assay. BT549 and MDA-MB-231 BC cell lines were pretreated with A009 extract (dilution 1:800) or Hyt (dilution of the major polyphenol present in the A009 extract, 1:800) for 24 h. The medium was then replaced with the chemotherapeutic drug Doxo 1 μM or 5-FU 100 μM, alone or in combination with the A009 extract or Hyt. Cells were treated for 48 and 72 h. The schedule of treatments and pretreatments is depicted in Supplementary Figure 2. Cell metabolic activity assessed by MTT of BT549 and MDA-MB-231 cells, receiving pretreatment with the extract A009 or Hyt, followed by the co-administration of these compounds with the chemotherapy drug 5-FU, was reduced compared with that of the cells receiving the 5-FU alone (Figures 2A,B). The same effect occurs on BT549 and MDA-MB-231 (Figure 3B), when treated with the A009 extract in combination with doxorubicin (Figures 3A,B). These results showed that the addition of chemotherapy to the A009 extract acts in an additive way reducing BC cell viability *in vitro*. We translated our results from a 2D to a 3D *in vitro* model of BC, by generating tumor spheroids, further treated following the same schedule applied for the 2D models. We observed that the combination of the A009 extract (1:800) with the chemotherapeutic agents 5-FU or Doxo synergized in blocking the generation of BC spheroids that morphologically appear less stable and with reduced diameter, within the time frame of treatments (Figures 4A–D).

### Cardioprotective Effect of A009 on Rat Cardiomyocytes

**Figure 5:**
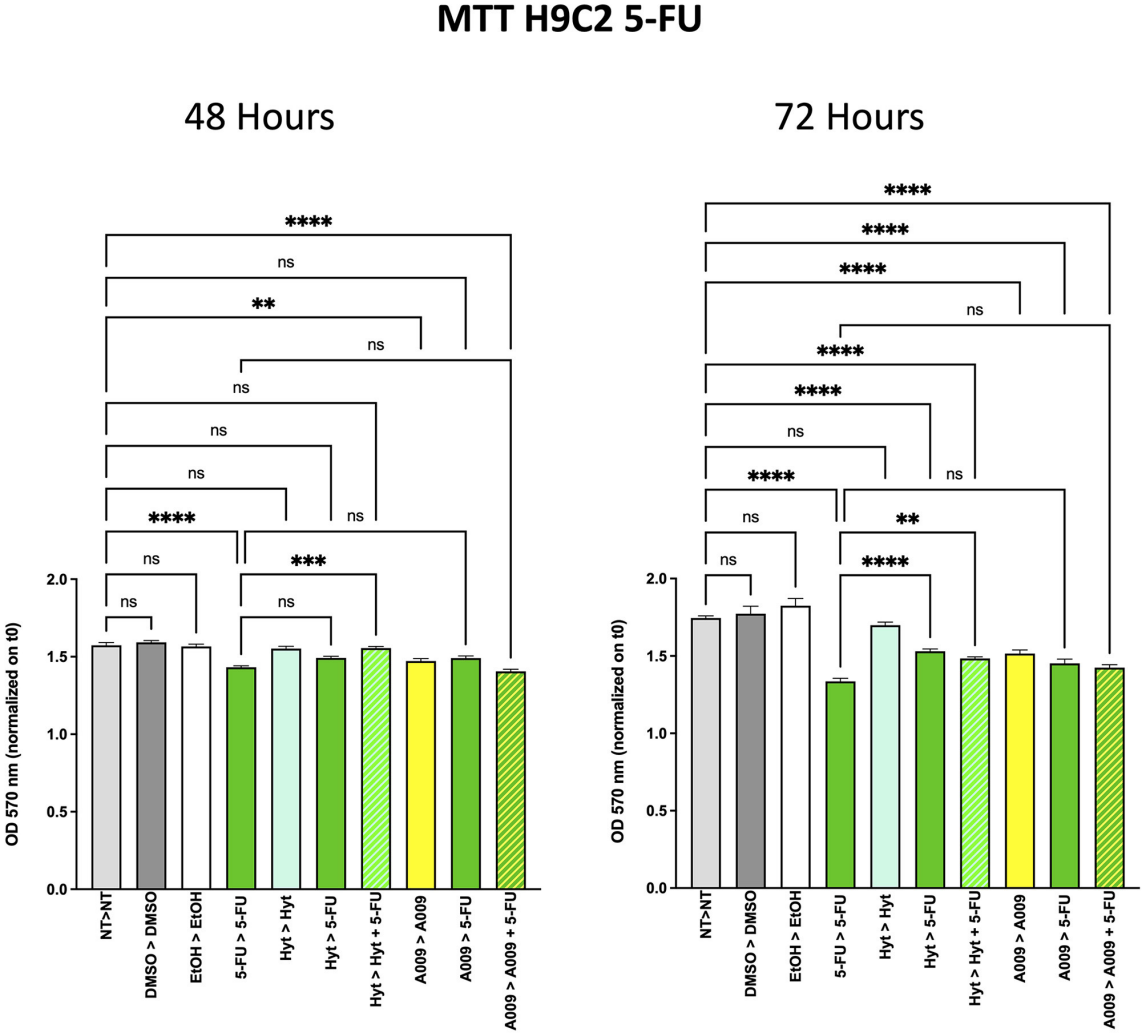
Effects of the A009 extract in combination with 5-FU on rat cardiomyocytes. H9C2 cells were pretreated with extract A009 (dilution 1:800) or Hyt (same concentration as that present in the 1:800 diluted A009 extract), for 24 h. Later, the medium was replaced with 5-FU 100 μM alone or in combination with A009 or Hyt for 48 and 72 h. Proliferation was detected by the MMT assay at the indicated time points. The experiments were performed in quadruplicate and repeated two times. Results are expressed as the mean of the absorbance normalized on the T0 ± SEM, one-way ANOVA, **p < 0.01, ***p < 0.001, and ****p < 0.0001. A009 combinations with chemotherapy were less toxic toward cardiomyocytes than toward breast cancer cells.

**Figure 6:**
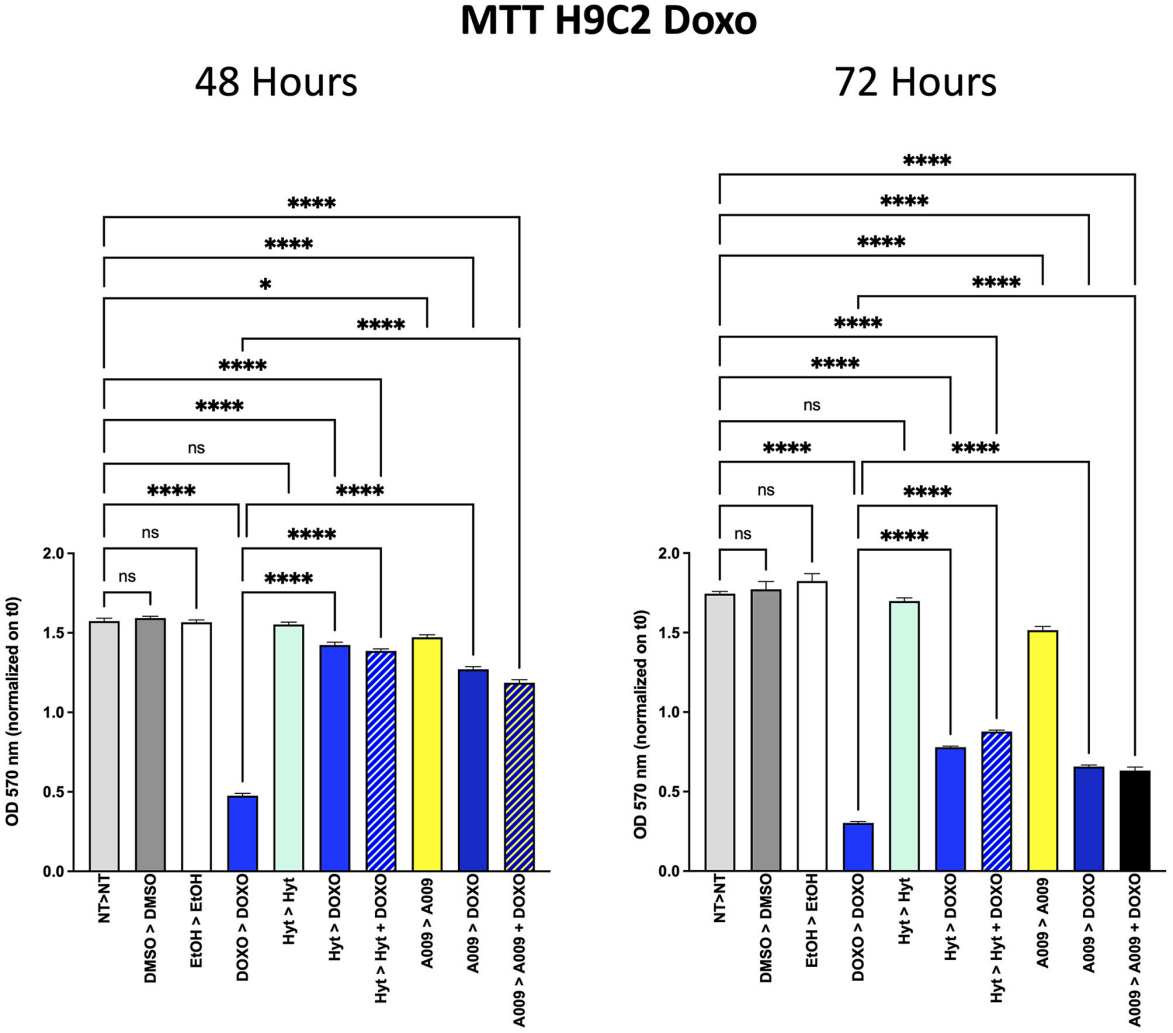
Effects of the A009 extract in combination with doxorubicin on rat cardiomyocytes. H9C2 cells were pretreated with extract A009 (dilution 1:800) or Hyt (same concentration as that present in the 1:800 diluted A009 extract), for 24 h. Later, the medium was replaced with and without Doxo 1 μM alone or in combination with A009 or Hyt for 48 and 72 h. Proliferation was detected by the MMT assay at the indicated time points. The experiments were performed in quadruplicate and repeated two times. Results are expressed as the mean of the absorbance normalized on the T0 ± SEM, one-way ANOVA, *p < 0.05 and ****p < 0.0001. A009 combinations with chemotherapy were less toxic toward cardiomyocytes than toward breast cancer cells.

Based on our previous published article on the cardioprotective properties of the A009 extracts against chemotherapy-induced damages in models of prostate cancers (11), we also tested whether a similar scenario could be observed with chemotherapeutic agents used in BC treatment, such as 5-FU and Doxo. We observed that the rat cardiomyocyte cell line H9C2 exhibited less reduced cell proliferation, when co-treated with the A009 + 5-FU (Figure 5) or A009 + Doxo (Figure 6), both at 48 and 72 h, as compared to 5-FU or Doxo alone. 5-FU and Doxo were toxic, while A009 or Hyt alone did not show cardiotoxicity. In addition, the co-administration of A009 or Hyt with the chemotherapy drugs does not increase damage induced by the drugs, Hyt + 5-FU after 48 and 72 h, and both Hyt + Doxo and A009 + Doxo after 48 and 72 h.

### Cardioprotective Effects of A009 Extract in Zebrafish Embryos

**Figure 7:**
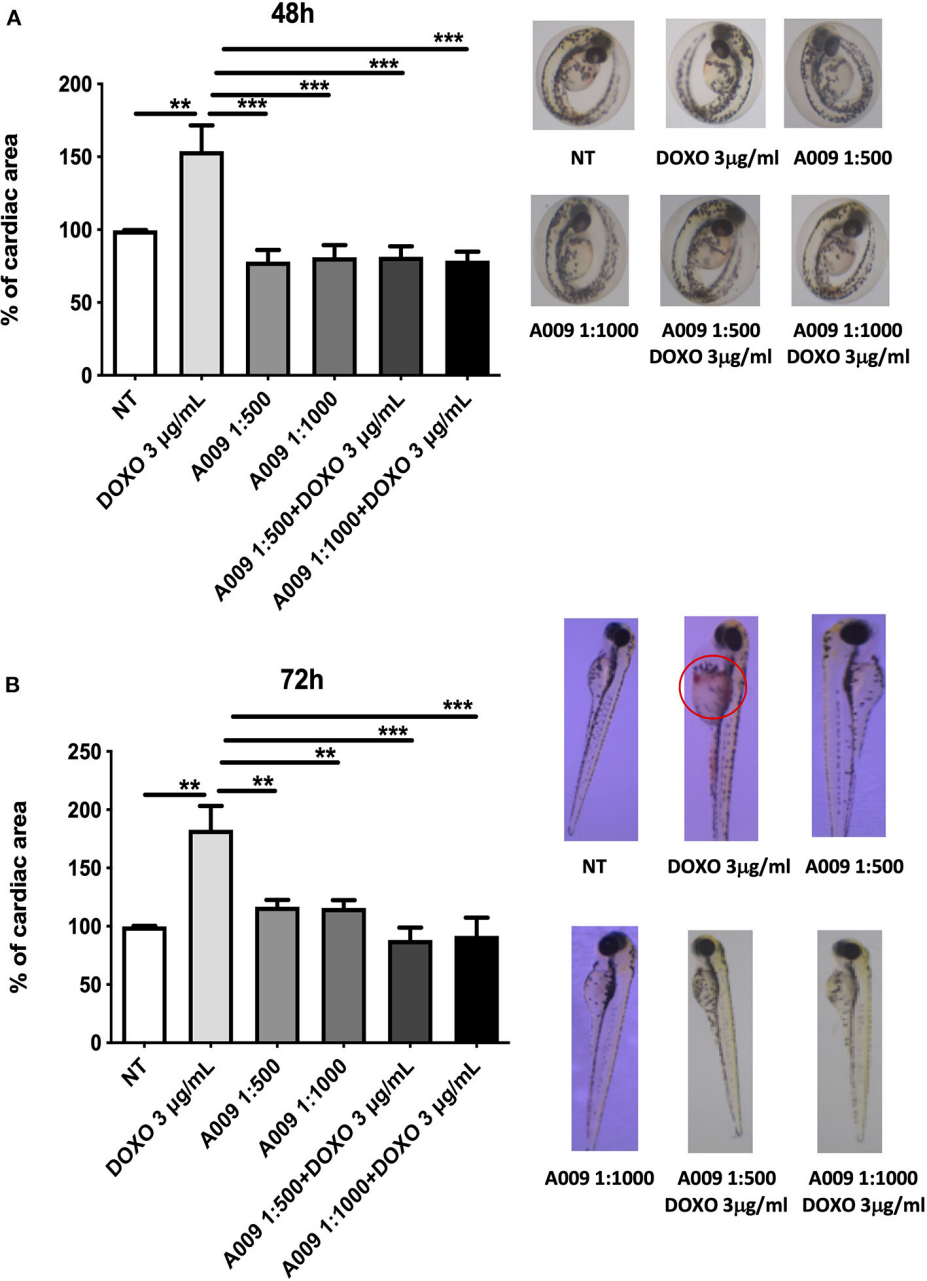
Cardioprotective effects of A009 extract in zebrafish embryos. The cardioprotective effects of the A009 extract on chemotherapy-induced cardiotoxicity was investigated in zebrafish embryos. All experiments were performed on zebrafish embryos exposed to doxorubicin (3 μg/ml) alone, A009 (dilution 1:1,000, 1:500), or the combination of doxorubicin (μg/ml) and A009 (dilution 1:1,000, 1:500) for 48 h **(A)** and 72 h **(B)** post fertilization (hpf). Embryo micrographs for all the experimental conditions are shown. Data for cardiac area are shown as fold% increased over control. Data are shown as mean ± SEM, one-way ANOVA, **p < 0.01 and ***p < 0.001. DOX, doxorubicin; A009 batch extract; NT, not treated. A009 combinations with chemotherapy were protective toward the heart.

We extended our *in vitro* results to the zebrafish (*Danio rerio*) animal model. Zebrafish embryos were exposed to Doxo, alone or in combination with the A009 extract (dilution 1:1,000 or 1:500). We observed that the treatment of zebrafish embryos with doxorubicin (3 g/ml) significantly reduced their cardiac area at 48 and 72 h of treatment (Figures 7A,B). The co-treatment with A009 was able to reverse the doxorubicin-induced cardiotoxic effect, in terms of cardiac area, following 48 and 72 h of treatment (Figures 7A,B). The polyphenolic concentrate alone does not change the viability of the embryos.

Furthermore, we observed that co-treatment of embryos with the A009 extract and doxorubicin resulted in decreased numbers of embryos displaying congenital abnormalities, when compared with embryos treated with doxorubicin alone (Supplementary Tables 3, 4).

### Effect of A009 on Inflammation and Induction of Senescence Associated With Chemotherapy in Human Cardiomyocytes

**Figure 8:**
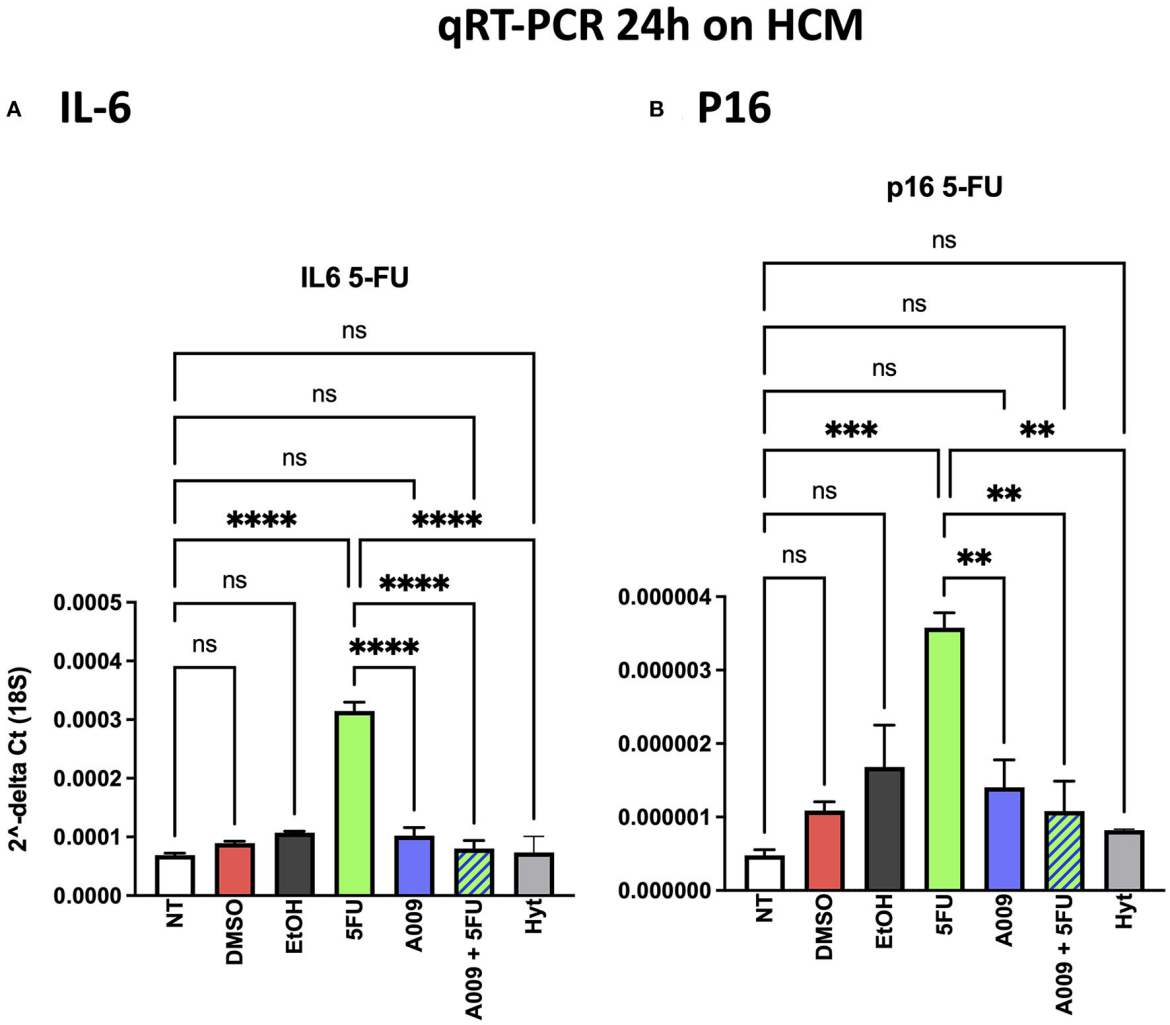
Effects of the A009 extract on interleukin (IL)-6 and p16 expression in human cardiac myocytes by qPCR. The ability of A009 (dilution 1:800) to inhibit **(A)** IL-6 and **(B)** p16 expression in HCM was determined, following 24 h of stimulation, by qPCR. Data are shown as mRNA relative expression, normalized to 18S and control, mean ± SEM, one-way ANOVA, **p < 0.01, ***p < 0.001, and **** *p* < 0.0001. A009 combinations with chemotherapy reduced IL6 and p16 expression.

Chemotherapy is often associated with damages to the heart that result in exacerbated cardiac inflammation and generation of senescent phenotype in cardiomyocytes. Il6 is among the cytokines involved in the cardiac inflammation and participating to the induction of the senescent-associated secretory phenotype (SASP), while p16 is a senescence marker. We investigated the effect of A009 on IL-6 and p16 gene expression, both molecules linking inflammation and senescence, following chemotherapy-induced (i.e., 5-FU) cardiac damages, on HCM cells. We found that HCM cells, exposed to the combination of A009 extract (1:800) and 5-FU 100 μM, exhibit decreased transcript levels of IL-6 (Figure 8A) and p16 (Figure 8B), as compared to HCM treated with 5-FU alone, in a statistically significant dependent manner.

## Discussion

Chemotherapy, alone or in combination with other therapies (i.e., targeted, antiangiogenic therapies), along with surgery and radiotherapy is still a major option in BC treatment. However, cancer chemotherapy-induced cardiotoxicity remains a relevant obstacle, thus limiting the therapeutic options (alone or in combination) for cancer patients (10, 35–37). This largely impacts on the management of oncologic patients, requiring more efforts to overcome the generation of side effects, following cancer chemotherapy that remains the treatment of election (10, 35–37). With the knowledge of such a relevant unmet clinical need, together with the identification of robust biomarkers able to predict chemotherapy-induced cardiovascular effects, prevention remains the most accessible option to manage such an issue (10, 35, 36), and cardio-oncology is a flourishing field of investigation (38, 39).

Polyphenols account as the major dietary-derived molecules endowed with beneficial effects on human health, based on their ability to target tumor cells, while sparing or recovering damaged normal/healthy cells. In this context, EVOO accounts as one of the most abundant dietary sources of polyphenols, within the Mediterranean diet. Interestingly, also the waste products derived by EVOO processing have been reported to be rich in polyphenols with beneficial health effects, such as hydroxytyrosol. We have previously demonstrated that the polyphenol-rich OMWW extract, A009, from EVOO is endowed with antiangiogenic properties and chemopreventive activities in the context of colon and prostate cancers, both *in vitro* and *in vivo*. Recently, we also showed, in *in vitro* and *in vivo* models of prostate cancer, that the combination of the A009 extract with chemotherapy resulted in increasing chemopreventive and antitumor activities, while mitigating the chemotherapy-induced damages to cardiomyocytes and mice hearts.

Starting from our previous results of antiangiogenic effects of the A009 extracts, in this study, we tested the ability of A009 to limit angiogenesis, a hallmark of cancer, *in vivo*, induced by CM from the BT549 BC cell line in a Matrigel sponge model that we contributed to develop (27–29). We found that the A009 extract limits angiogenesis *in vivo*, induced by factors present in the BT549 cell CM. We also observed that the BT459 CM sponges from mice treated with the A009 extract show increased infiltration of CD3^+^T cells, suggesting a potential contribution of the OMWW extract in the recruitment of immune cells.

We then tested the capability of the combination of the A009 extract with chemotherapeutic agents clinically employed in BC, to act on BC cell proliferation. We found that both the BT549 and MDA-MB-231 cells, following exposure to the A009 + 5-FU or A009 + Doxo combination, exhibited reduced cell proliferation, as compared with those treated with A009 alone. We observed additive effects by treating BT459 and MDA-MB-231 cell spheroids, with the A009 + drug combination. These results show that the combination of chemotherapy with the A009 extract further reduces BC cell viability *in vitro*.

A peculiar capability of polyphenols resides in their ability to target transformed malignant cells (40) also cooperating with chemotherapeutic agents (11, 40, 41), while sparing healthy cells or recovering healthy cells undergoing stress conditions and cellular damages (42–44). Based on this evidence and on our previous published article on the cardioprotective properties of the A009 extract against chemotherapy-induced damages, in models of prostate cancers, we tested whether a cardio-oncological prevention scenario could be observed, using chemotherapeutic agents used for BC treatment, such as doxorubicin and fluoropyrimidines. The combination of A009 extract to chemotherapy mitigate the effects of reduced cell proliferation, mediates by 5-FU alone on rat cardiomyocytes. We found that this cardioprotective effect of the A009 extract occurs also *in vivo*: zebrafish embryos exposed to A009 extract + Doxo combination show rescue of cardiac area, as compared with those treated with Doxo alone.

OMWW extracts are not toxic to the animals, and in a cohort of healthy, individuals were highly tolerated with no toxicity (11).

Inflammation represents a peculiar hallmark of chronic diseases, such as cancer, metabolic, and cardiovascular disorders (45–48). Chemotherapy-induced cardiovascular side effects also include exacerbated inflammation, together with induction of cell senescence and of an SASP. We previously demonstrated that cardiovascular toxicities associated with the anticancer agent 5-FU include the induction of a senescent phenotype in HCMs and endothelial cells (49).

IL-6 accounts as a relevant cytokine in the inflammatory process (50) and is highly represented in the cytokine milieu characterizing the SASP phenotype (51, 52). In line with this evidence, we found that HCMs treated with 5-FU, have increased transcript levels of IL-6 and the senescence marker p16. We observed that the combination of the A009 extract with 5-FU can reduce the expression level of IL-6 and p16, induced by the 5-FU. This suggests the potential capability of the A009 extracts to exert cardioprotective activities also acting on the inflammation/senescence pathways in cardiomyocytes exposed to chemotherapeutic agents.

## Conclusion

Our study suggests that in a cardio-oncological prevention perspective, a polyphenol-rich purified OMWW extract A009 combined with cancer chemotherapy, could represent a potential candidate for cardiovascular protection in patients with BC, while increasing effects of BC chemotherapy.

## Data Availability Statement

The raw data supporting the conclusions of this article will be made available by the authors, without undue reservation.

## Ethics Statement

All the procedures applied were approved by the Local Animal Experimentation Ethics Committee (ID# #06_16 Noonan) of the University of Insubria and by the Health Ministry (ID#225/2017-PR).

## Author Contributions

DN, AB, RR, and AA: conceptualization, writing revision, and data curation. AB, DN, and AA: formal analysis and supervision. DN and AA: funding acquisition and project administration. NB, LC, KG, MGC, GP and AB: methodology. NB and LC: writing—original draft. GP and MGC: writing revision. All authors contributed to the article and approved the submitted version.

## Funding

This work has been supported by Italian Ministry of Health Ricerca Corrente—IRCCS MultiMedica.

## Conflict of Interest

The authors declare that the research was conducted in the absence of any commercial or financial relationships that could be construed as a potential conflict of interest.

## Publisher's Note

All claims expressed in this article are solely those of the authors and do not necessarily represent those of their affiliated organizations, or those of the publisher, the editors and the reviewers. Any product that may be evaluated in this article, or claim that may be made by its manufacturer, is not guaranteed or endorsed by the publisher.

## Supplementary Material

Supplementary Figure 1 Macrophages and natural killer (NK) cells in the UltiMatrix sponge assay. Changes in macrophages and NK cells upon treatment are nonsignificant.

Supplementary Table 1 Phenolic composition of A009 obtained by high-performance liquid chromatography coupled with mass spectrometry (HPLC-DADMS-MS). Samples were analyzed by HPLC with UV-vis and MS detection. The identification of phenolic compounds from samples was carried out as previously reported by interpreting their mass spectra determined *via* LC-MS-MS and comparing with the data reported in the literature.

Supplementary Table 2 Treatment's scheme of 3-(4,5-dimethylthiazol-2-yl)-2,5-diphenyltetrazolium bromide (MTT) assay on BT549, MDA-MB-231, and H9C2 cell lines. Cells were treated with A009 extract (dilutions 1:800) and Hyt (dilution 1:800) for 24 h. The medium was then substituted with the chemotherapy drug 5-FU 100 μM or Doxo 1 μM, alone or in combination with A009 or Hyt for 48 and 72 h.

Supplementary Tables 3, 4 Presence of congenital alterations in zebrafish embryos, during single agent (A009, dilution 1:1,000, 1:500) alone, doxorubicin (3 μg/ml) alone, or the combination of doxorubicin and the A009 extract treatments, was monitored. Data are presented as the frequency (%) of events per experimental condition, at 24 **(A)** and 48 **(B)** h of treatment. Ischemia in Yolk Sack (IS-YS); malformation of heart (M.HT); ischemia in the tail (IS-TA); malformation of the tail (MT); yolk sack malformation (YS-DE); swim bladder malformation (SWB-DE); pericardial edema (PE); ischemia in the brain (IS-BR). Congenital alterations are mitigated by A009.
