# Supplementary material for: An Olive Oil Mill Wastewater Extract Improves Chemotherapeutic Activity Against Breast Cancer Cells While Protecting From Cardiotoxicity
Source: Front Cardiovasc Med. 2022 Apr 14;9:867867. doi: 10.3389/fcvm.2022.867867 (PMC9047943; doi:10.3389/fcvm.2022.867867)
Supplement: Supplementary Figure 1 — Macrophages and natural killer (NK) cells in the UltiMatrix sponge assay. Changes in macrophages and NK cells upon treatment are nonsignificant. [file Data_Sheet_1.PDF]

Supplemental Table S1

PHENOLIC COMPOUND	A009 (g/L)
Hydroxytyrosol glucoside	1,69
Hydroxytyrosol	5,72
Tyrosol	ND
Chlorogenic acid	0,10
b-hydroxyverbascoside isomer 1	0,14
b-hydroxyverbascoside isomer 2	0,17
Verbascoside	1,32
Caffeoyl ester of secologanoside	0,20
Decarboxymethyloleuropein aglycon	0,28
Oleuropein aglycon	0,22
6’-p-coumaroyl secologanoside	0,40
Rutin	ND
Luteolin-7-o-glucoside	ND

Supplemental table S2

Treatment schedule MTT BT549, MDAMB231, H9C2

	T1 (24h)	T2 (48h)	T3 (72h)
**1**	NT	NT	NT
**2**	DMSO	DMSO	DMSO
**3**	EtOH	EtOH	EtOH
**4**	5-FU	5-FU	5-FU
**5**	DOXO	DOXO	DOXO
**6**	Hyt	Hyt	Hyt
**7**	Hyt	5-FU	5-FU
**8**	Hyt	DOXO	DOXO
**9**	Hyt	Hyt + 5-FU	Hyt + 5-FU
**10**	Hyt	Hyt + DOXO	Hyt + DOXO
**11**	A009	A009	A009
**10**	A009	5-FU	5-FU
**11**	A009	DOXO	DOXO
**12**	A009	A009 + 5-FU	A009 + 5-FU
**13**	A009	A009 + DOXO	A009 + DOXO

Supplemental figure 1

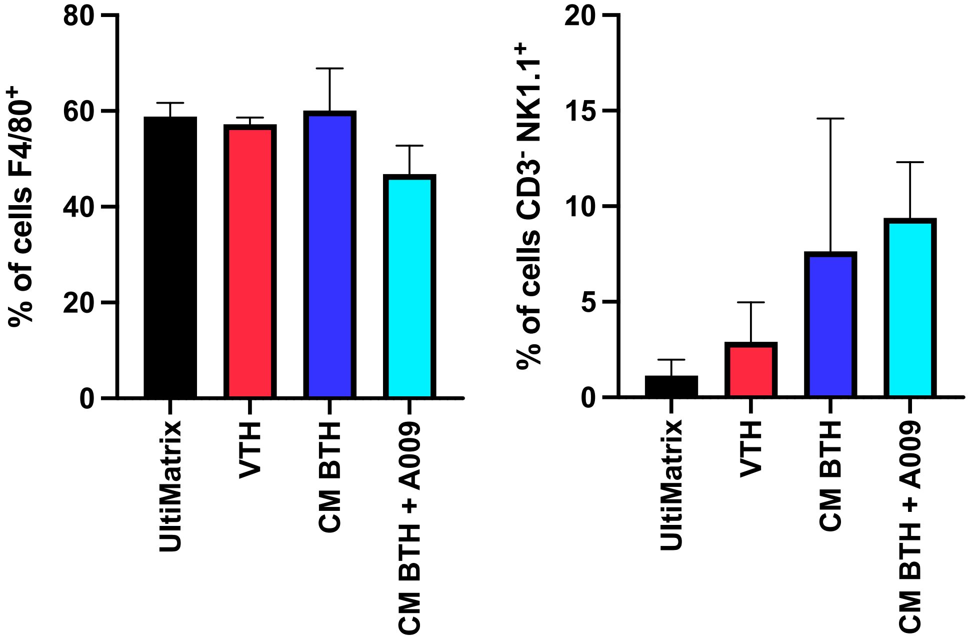

Supplemental Table S3

TYPE OF MALFORMATION AT 24 hpf	NT	Doxo	A009 1:500	A009 1:1000	A009 1:500 + Doxo	A009 1:1000 + Doxo
IS-YS	0	0,7	0	0	0,3	0,5
M.HT	0%	60%	0%	0%	40%	40%
IS-TA	0%	60%	0%	0%	20%	20%
MT	0%	20%	0%	0%		0%
YS-DE	0%	50%	0%	0%	10%	20%
SWB-DE	0%	70%	0%	0%		10%
PE	0%	70%	0%	0%	40%	60%
IS-BR	0%	60%	0%	0%	10%	0%

Supplemental Table S4

TYPE OF MALFORMATION AT 48 hpf	NT	Doxo	A009 1:500	A009 1:1000	A009 1:500 + Doxo	A009 1:1000 + Doxo
IS-YS	0	0,8	0	0	0,4	0,6
M.HT	0%	50%	0%	0%	40%	30%
IS-TA	0%	70%	0%	0%	30%	40%
MT	0%	30%	0%	0%	20%	0%
YS-DE	0%	30%	0%	0%	0%	10%
SWB-DE	0%	60%	0%	0%	40%	50%
PE	0%	100%	0%	0%	60%	70%
IS-BR	0%	20%	0%	0%	10%	0%
